# Cannabis use in Patients With Inflammatory Bowel Disease is Associated With Longer Endoscopic Duration and Endoscopic Inflammation

**DOI:** 10.1093/crocol/otaf034

**Published:** 2025-07-02

**Authors:** Lauren Loeb, Alexander Hochwald, Michael F Picco, Johanna L Chan, Jana G Hashash, Ryan Chadha, Francis A Farraye, Jami A Kinnucan

**Affiliations:** Department of Gastroenterology and Hepatology, Mayo Clinic, Jacksonville, FL, USA; Clinical Trials and Biostatistics, Mayo Clinic, Jacksonville, FL, USA; Department of Gastroenterology and Hepatology, Mayo Clinic, Jacksonville, FL, USA; Department of Gastroenterology and Hepatology, Mayo Clinic, Jacksonville, FL, USA; Department of Gastroenterology and Hepatology, Mayo Clinic, Jacksonville, FL, USA; Department of Anesthesiology, Mayo Clinic, Jacksonville, FL, USA; Department of Gastroenterology and Hepatology, Mayo Clinic, Jacksonville, FL, USA; Department of Gastroenterology and Hepatology, Mayo Clinic, Jacksonville, FL, USA

**Keywords:** inflammatory bowel disease, cannabis, endoscopy, propofol

## Abstract

**Background:**

Patients with inflammatory bowel disease (IBD) often experience symptoms refractory to available treatments, prompting the use of alternative therapies like cannabis. Previous studies have shown cannabis users require higher levels of sedation for procedures. The anti-inflammatory effects of cannabis have been studied with mixed conclusions. We aimed to investigate if patients with IBD who reported cannabis use required more resources for endoscopic procedures and were more likely to have endoscopic inflammation.

**Methods:**

This is a retrospective case–control study of adult patients with IBD between November 2018 and November 2022 at a tertiary academic medical center undergoing endoscopic evaluation of acute complaints related to IBD. Cases were matched for age, sex, and body mass index.

**Results:**

There were 124 patients with IBD with 62 patients reporting cannabis use and 62 patients without reported cannabis use. There was a significant difference in endoscopy duration (*P* < .001) and endoscopic inflammation (*P* = .044) between groups. There was no significant difference in recovery room length of stay (*P* = .15), IBD treatment during time of endoscopy (*P* = .84), stricture (*P* = .53), propofol dose administered when adjusted for procedure duration (*P* = .082), or endoscopic duration between cannabis users with and without endoscopic inflammation (*P* = .194).

**Conclusions:**

IBD cannabis users had longer endoscopic durations and were more likely to exhibit inflammation on endoscopic evaluation compared to cannabis non-users. Our study underscores the importance of medication reconciliation for more accurate resource allocation. Additionally, federal expansion of cannabis research is needed for randomized control trials to fulfill the presently unmet need for data on patient outcomes.

## Graphical Abstract

**Graphical Abstract:**
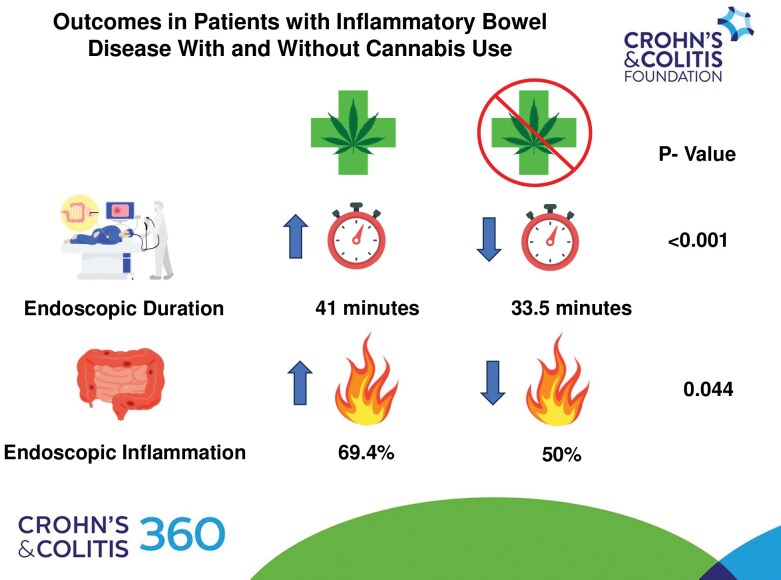

**Key Messages**

• What is already known?
- Patients with IBD often experience nausea, abdmoninal pain, urgency, and diarrhea refractory to available therapies.
• What is new here?
- Patients with IBD in our cohort who used cannabis had longer endoscopies and more frequent evidence of inflammation on endoscopy than those without reported cannabis use.
• How can this study help patient care?
- This study emphasizes the importance of medication reconciliation before endoscopic evaluation and the need for randomized control trials on the clinical outcomes of cannabis use.

## Introduction

Inflammatory bowel disease (IBD), including Crohn’s disease (CD) and ulcerative colitis (UC), is a chronic condition characterized by inflammation of the gastrointestinal (GI) tract that requires chronic medical management and sometimes surgical intervention. Patients with IBD often experience symptoms such as abdominal pain or cramping, diarrhea, and decreased appetite. These symptoms can result in a diminished quality of life that leaves patients eager for interventions that will ameliorate these symptoms.^1^ IBD management has improved over the last decade with the introduction of advanced therapies^2^; however, despite the growing number of options to treat patients with IBD, there is a significant population of patients with IBD who experience symptoms refractory to current medical management.^3,4^

In addition to the increased use of advanced therapies, there has been increasing legislation around cannabis legalization. California was the first state to legalize medical cannabis use in 1996. As of 2022, cannabis is legal for medical use in 18 states and is legal for both medical and recreational use in an additional 18 states and Washington D.C.^5^ With cannabis becoming more accessible, patients are increasingly trialing cannabinoid products to investigate the benefits it may offer.^6^ Patients with IBD are no exception.^7^ Due to the mechanism of cannabis in the endocannabinoid system, we see clinical effects on pain, nausea, diarrhea, and appetite.^8,9^ However, it is challenging to study cannabinoids given the current federal legalization status, the inability to truly blind those exposed to cannabis, and the variability in cannabis strains, formulations, and routes of administration.^10^

The analgesic properties that make cannabis attractive to patients with IBD are mediated by the same receptor that gives propofol its sedative effect.^11^ The most well-known of the cannabinoids are delta-9-tetrahydrocannabinol (THC) and cannabidiol (CBD). Receptors for these cannabinoids are found throughout the body, including the nervous system, GI tract, and certain immune cells.^12^ Most notable among the cannabinoid receptors are cannabinoid receptors 1 and 2 (CB1, CB2).^13^ Both synthetic and endogenous cannabinoids bind to the CB1 and CB2 receptors to produce the analgesia associated with cannabis use.^14^ Previous studies have found that activation of CB1 as well as GABA_A_ are required to produce the sedative effect of propofol.^15^ Although propofol does not act directly on CB1, it does increase brain levels of anandamide, an endogenous cannabinoid, that directly activates CB1.^15^ This commonality between propofol and cannabis raises a question of upregulation of endogenous cannabinoids by propofol with the use of cannabis.

Given the increasing use of cannabis in the United States and among patients with IBD,^16^ we sought to evaluate for potential implications of cannabis use on healthcare resources and IBD activity. We hypothesized that patients with IBD who use cannabis are more likely to require higher doses of propofol and have longer endoscopic procedure time and length of recovery than patients with IBD who do not report cannabis use. Additionally, we hypothesized those who reported cannabis use would have a higher incidence of endoscopic inflammation than those who did not report cannabis use.

## Material and methods

A retrospective case–control study of patients with IBD undergoing either colonoscopy (COL), esophagogastroduodenoscopy (EGD), or both for evaluation of acute symptoms related to IBD was conducted at a tri-state tertiary academic medical center. Patients were excluded from the study if they reported daily alcohol, opioid, or benzodiazepine use during pre-procedural evaluation by either a gastroenterology provider or anesthesiologist. Those with medical diagnoses of cirrhosis, chronic kidney disease, obesity, or pregnancy were also excluded. Between November 2018 and November 2022, adult patients with IBD were identified who met the above criteria and reported cannabis use. Cannabis use was defined as patients with any duration of self-reported cannabis use within one month of endoscopy at either the clinic visit before the endoscopy or the pre-procedure medication reconciliation with the anesthesiologist (cannabis users). This group was matched for age, sex, and body mass index (BMI) to patients with IBD who did not report cannabis use (cannabis non-users).

Several variables were collected on review of the electronic medical records, including the type of endoscopy (COL, EGD, or both), duration of endoscopy (minutes), length of stay in the recovery room (minutes), propofol bolus dose to initiate sedation in milligrams (mg), total propofol dose administered during endoscopy (mg), and the name of the endoscopist. Due to a lack of consistency in the choice of endoscopic scoring systems, the activity of IBD was evaluated by reading the report of each endoscopy for language indicative of mucosal inflammation and stricture. Additionally, images associated with the endoscopy were reviewed for gross inflammation. Active use of IBD therapy during the time of endoscopy was determined by reviewing notes within one month of endoscopy. IBD therapies identified included biologics (infliximab, adalimumab, certolizumab, vedolizumab, ustekinumab, risankizumab, guselkumab), aminosalicylates (sulfasalazine, mesalamine), immunomodulators (azathioprine, mercaptopurine, methotrexate, cyclosporine), and steroids.

Demographic information was collected, including sex, age at endoscopy, BMI at endoscopy, and IBD diagnosis (CD or UC). Continuous variables were compared with a Kruskal–Wallis test and categorical variables were compared with Fisher’s exact test. All statistical tests were 2-sided. *P*-values ≤.05 were considered statistically significant.

The primary endpoints evaluated were the dose of propofol administered during endoscopy, duration of endoscopy, length of stay in the recovery room, endoscopic inflammation and stricture, and IBD medication use at the time of endoscopy. Secondary outcomes included temporal and financial implications of the primary data. The study protocol was approved by the Institutional Review Board (22-011742).

## Results

A total of 62 patients were identified as meeting our inclusion criteria and an additional 62 matched controls were included. Demographic characteristics for all patients are presented in Table 1. Of the 124 patients, 60 had a diagnosis of CD, and 64 had a diagnosis of UC. Thirty (50%) of the patients with CD and 32 (50%) of the patients with UC reported cannabis use. The average age, sex, and BMI were similar among both groups. In both groups, the most common endoscopic procedure was COL alone (71.0% and 79.0%, respectively), followed by EGD alone (16.1% and 11.3%, respectively) and both COL and EGD (12.9% and 9.7%, respectively).

**Table 1.** Patient Demographics

	No Cannabis use		Cannabis use	
Variable	*N*	Median (minimum, maximum) or No. (%) of patients	*N*	Median (minimum, maximum) or No. (%) of patients
Age at endoscopy	62	40 (18, 72)	62	36 (18, 74)
Sex (male)	62	34 (54.8%)	62	34 (54.8%)
BMI at procedure	62	24.4 (16.2, 29.8)	62	24.3 (17.2, 29.9)
IBD type	62		62	
Crohn’s disease		30 (48.4%)		30 (48.4%)
Ulcerative Colitis		32 (51.6%)		32 (51.6%)
Endoscopy type	62		62	
Colonoscopy alone		49 (79.0%)		44 (71.0%)
EGD alone		7 (11.3%)		10 (16.1%)
Colonoscopy and EGD		6 (9.7%)		8 (12.9%)

An analysis of cannabis use as it relates to resources utilized during endoscopy is provided in Table 2. Although there was a significant difference between cannabis users and cannabis non-users in the dose of propofol administered during all endoscopic procedures (*P* < .001) when controlling for the duration of endoscopy, there was not a significant difference in the amount of propofol administered (*P* = .082). There was a significant difference in the duration of endoscopy of any type (COL, EGD, or both) between the 2 groups (*P* < .001). Cannabis users had a median duration of 41 minutes (range 18-92 minutes) compared to 33.5 minutes (range 16-92 minutes) in the cannabis non-users. There was no significant difference found in the amount of time spent in the recovery room after endoscopy (*P* = .15); or the dose of propofol bolus required to initiate sedation (*P* = .18).

**Table 2.** Analysis of Resources by Reported Cannabis Use

	No Cannabis use		Cannabis use		*P*-value
Variable	*N*	Median (minimum, maximum)	*N*	Median (minimum, maximum)	
Total Propofol administered (mg)	62	298.8 (100, 755)	62	444 (132.1, 1200)	<.001
Total Propofol administered (mg) colonoscopy only	49	299.7 (100, 712.9)	44	444 (156.2, 1200)	<.001
Duration of endoscopy (minutes)	62	33.5 (16, 92)	62	41 (18, 92)	**<.001**
Propofol (mg)/minute	62	8.8 (2.8, 25.5)	62	9.8 (3.8, 31.6)	.082
Recovery room length of stay (minutes)	62	37 (21, 114)	62	42.5 (21, 160)	.15
Propofol Bolus to initiate sedation (mg)	62	70 (20, 200)	62	80 (10, 270)	.18

Data used to evaluate the activity of IBD is included in Table 3. Of the cannabis users, 69.4% were found to have endoscopic inflammation compared to 50% of cannabis non-users (*P* = .044). There was no significant difference in the use of IBD therapy at the time of endoscopy (*P* = .84) or frequency of stricture (*P* = .53) between groups.

**Table 3.** Inflammatory Bowel Disease Activity and Treatment

	Cannabis use (*N* = 62)	No Cannabis use (*N* = 62)	Total (*N* = 124)	P value
Evidence of endoscopic inflammation				**.044**
No	19 (30.6%)	31 (50.0%)	50 (40.3%)	
Yes	43 (69.4%)	31 (50.0%)	74 (59.7%)	
Was the patient on IBD treatment during endoscopy				.84
No	18 (29.0%)	16 (25.8%)	34 (27.4%)	
Yes	44 (71.0%)	46 (74.2%)	90 (72.6%)	
Stricture noted on endoscopy				.53
No	55 (88.7%)	58 (93.5%)	113 (91.1%)	
Yes	7 (11.3%)	4 (6.5%)	11 (8.9%)	

Table 4 reports findings on evidence of endoscopic inflammation in cannabis users in the context of the duration of endoscopy. There was not a significant difference in the duration of endoscopy between cannabis users who were found to have endoscopic inflammation and those who did not have evidence of endoscopic inflammation (*P* = .194).

**Table 4.** Endoscopy Duration in Cannabis Users With and Without Inflammation

	Cannabis Use Duration (minutes) *P* value (*N* = 62)		
Variable	No. (%) of patients	Median (minimum, maximum)	
No endoscopic inflammation	19 (30.6%)	41 (22.0, 59.0)	.194
Yes endoscopic inflammation	43 (69.4%)	42 (18.0, 92.0)	

## Discussion

As the stigma and legal boundaries once associated with cannabis use continue to decrease, it is important to recognize the implications that arise for both patient safety and outcomes as well as healthcare resource allocation. We found that IBD cannabis users required higher doses of propofol and longer endoscopic procedures compared to IBD cannabis non-users. The higher propofol doses and longer duration of endoscopy in cannabis users in our cohort raise the question of whether the higher sedation requirements were simply due to longer procedures. This was evaluated by comparing the propofol doses between groups when adjusted for the duration of endoscopy. There was not a significant difference in mg of propofol per minute (*P* = .082), suggesting the higher propofol requirements in cannabis users were likely a product of longer time under sedation rather than metabolically mediated.

Despite the paucity of standardized research regarding cannabis, there have been several studies that comment on the administration of propofol in the setting of cannabis use. Brand and colleagues^17^ found the sedative effect of a low propofol concentration in mice was eliminated by analgesic doses of THC; however, increasing propofol concentrations ultimately established sedation despite THC. Imasogie and colleagues^18^ conducted a case–control study in Canada that found higher doses of propofol were required for sedation in patients who reported cannabis use compared to those who denied cannabis use, with the highest doses required by daily cannabis users. Similarly, Kosirog and colleagues^19^ performed a prospective case–control study at the Oklahoma City Veterans Affairs Medical Center that found cannabis use to be an independent predictor for higher propofol requirement during routine endoscopy. Unlike our study, there was not a statistically significant difference in procedure duration between groups in either study by Imasogie and colleagues or Kosirog and colleagues. This reintroduces the question of potential metabolic consequences of cannabis use on propofol dosing requirements. Although the exact interaction between propofol and cannabinoids is presently unknown, there are hypotheses that suggest a pharmacokinetic explanation.^20^ For example, both propofol and THC are highly lipophilic and have long half-lives.^21–23^ In patients who use THC, these factors may produce an interaction when such patients receive propofol resulting in tachyphylaxis and the requirement of higher doses to achieve sedation. Another theory notes the common receptor that both propofol and cannabis act upon: GABA_A_. The possibility of a CB1 and GABA_A_ receptor complex may lower GABA neurotransmission and result in higher propofol requirements.^22^ Twardowksi and colleagues^14^ performed a retrospective study that found the 25 cannabis users required 220.5% more propofol to achieve continuous sedation during endoscopy than the 225 cannabis non-users. This study did not report the duration of endoscopy to allow investigation into whether higher propofol doses were due to longer duration. King and colleagues^21^ investigated propofol requirements for patients undergoing EGD in 47 cannabis users and 23 cannabis non-users. No statistically significant differences were found in propofol requirements between groups. It should be noted that none of these studies examine patients with IBD specifically. Ours is the only study to our knowledge to date that investigates propofol requirements in patients with IBD who report cannabis use.

We found that IBD cannabis users were more likely to exhibit inflammation on endoscopy. The role of cannabis in the management of inflammation and associated symptomology of IBD has been studied with mixed conclusions. Anti-inflammatory properties of cannabis have been described through cannabinoid-mediated actions on adenylyl cyclase, beta-gamma pathways, and intracellular free calcium levels.^24^ These properties were evaluated clinically by a prospective case–control study by Naftali and colleagues^25^ when 21 patients with CD and CDAI scores greater than 200 with disease refractory to steroids, immunomodulators, and antitumor necrosis factor agents were randomized into either the treatment THC cigarette group or placebo cigarette groups. Clinical response, defined by a decrease in CDAI score of greater than 100, was observed in 90% of the subjects in the treatment group compared to 40% of subjects in the placebo group (*P* = .03). Importantly, this study addresses subjective, symptomatic improvement only. Changes in C-reactive protein (CRP) were not appreciated, suggesting the improvement in CDAI may be secondary to the central effects of cannabis rather than mitigation of inflammation. Similarly, Irving and colleagues^26^ performed a randomized, double-blind, placebo-controlled, parallel-group pilot study assessing the efficacy of an oral CBD-rich extract on remission rates in patients with UC. Although there was no difference in remission rates between the treatment and placebo groups, the treatment group had significant improvement in the patient-reported global impression of change (*P* = .003). Both of these studies support our finding that cannabis use likely does not improve objective mucosal inflammation in patients with IBD.

Interestingly, there was not a significant difference in the use of medical IBD therapies between cannabis users and cannabis non-users. We initially hypothesized that this variable would differ between groups, as noncompliance with medical treatment would likely predispose the patient to worse disease and potentially prompt alternative means of symptom control such as cannabis. One possible explanation for the discrepancy in endoscopic inflammation between groups despite similar rates of medical IBD treatment may paradoxically be due to the symptomatic improvement cannabis can provide. A retrospective study by Glickman and colleagues found IBD cannabis users demonstrated an increased risk for requiring corticosteroids (risk ratios [RR], 1.095; 95% CI, 1.021-1.174; *P* = .011), emergency department visits (RR, 2.143; 95% CI, 2.034-2.257; *P < .*001), and hospitalizations (RR, 1.925; 95% CI, 1.783-2.079; *P < .*001).^27^ This raises concern for a potential masking effect cannabis may have on IBD-related inflammation that may result in delayed care and worse outcomes.^28^ Consideration was given to evaluating for differences in the exhibition of inflammation between patients on various classes of IBD therapy (biologic, aminosalicylates, immunomodulators, steroids); however, given the lack of statistically significant differences appreciated in the use of IBD therapies between cannabis users and cannabis non-users, the conclusion was drawn that further stratification by type of IBD therapy would produce a similarly insignificant result.

In order to identify a possible explanation for the significantly longer duration of endoscopy in cannabis users compared to cannabis non-users, the duration of endoscopy in cannabis users who were found to have endoscopic inflammation was compared to cannabis users. Our hypothesis was that cannabis users with endoscopic inflammation would have longer endoscopic durations, potentially pointing towards the presence of inflammation as the reason for the difference in endoscopic duration between cannabis users and non-users. However, there was not a significant difference in endoscopic duration between cannabis users with and without inflammation, suggesting that the presence of inflammation alone is unlikely to explain differences in the duration of endoscopy. This analysis leads to further questions rather than an answer, further underscoring the need for randomized control studies on the outcomes associated with cannabis use. With reclassification to Schedule III status under the Controlled Substance Act, these studies might be more feasible.

Consistent with the existing literature, our findings emphasize the importance of identifying all therapeutic agents, both prescribed and not prescribed, used by patients. The initial office visit with patients with IBD should include a thorough medication reconciliation and substance abuse history to optimally identify the appropriate level of sedation for prospective endoscopies. Additionally, this will allow for a more accurate estimation of the time needed to complete an endoscopy. The higher incidence of endoscopic inflammation appreciated in IBD cannabis users may reflect a propensity to pain that may have contributed towards the significantly longer endoscopic duration in these patients. This information can help practices avoid the extra cost associated with procedures that extend past the assigned hours.

Although the average difference in the median duration of endoscopy between IBD cannabis users and cannabis non-users was only 7 and a half minutes, the cost accrued during this time is worth noting. This is especially true among these patients who more frequently have commercial insurance as opposed to Medicare. Across the 3 academic medical centers in which the endoscopies in this study were performed, the IBD procedure room is open 480 minutes per day. Considering the professional and facility fees, it is estimated that each procedure room generates 20 000 dollars daily; therefore, 312.50 dollars is generated in 7 and a half minutes. Compounded over even a handful of patients, this presents a significant financial burden if not accounted for preemptively. Additionally, these delays in patient turnover can result in changes to endoscopy throughput and the ability to attend to patients in an organized system of care.

One limitation of this study is the retrospective design, which may introduce biases in the data collection. This design restricted the ability to collect certain data, including labs to corroborate adherence to prescribed IBD treatments, urine drug tests to confirm recent cannabis use and frequency, dose, and route of administration of cannabis use. However, it should be noted that even if this were a prospective study, current quantitative cannabis testing is not sensitive or specific enough to confirm or negate recent cannabis use due to the nonlinear relationship between plasma or urine cannabinoid levels and degree of intoxication.^29^ Additionally, the retrospective design prevented the implementation of a standardized endoscopic scoring system to more reliably characterize the degree of inflammation. The 124 endoscopies included in our study were done across 3 campuses by 82 endoscopists resulting in appreciable variety in the type and degree of detail documented regarding endoscopic inflammation. This led to our binary method of evaluating the presence of inflammation in order to prevent or at least limit inaccurate, biased, or subjective interpretations. It should also be noted that documentation of NSAID use or inclusion of these agents in the medication list of patients in our cohort was minimal. This is likely due to the perspective of some patients that over-the-counter therapies like NSAIDs are conceptualized differently than those that are prescribed by a healthcare provider, resulting in possible under-reporting of their use. This introduces the possibility that endoscopic inflammation may be secondary to or contributed to by unidentified NSAID use.

The modest sample size may affect the power to detect associations and thus present the possibility of a type II error. A substantial contributor to propofol administration during endoscopy is the comfort of the endoscopist with various levels of sedation. For example, some endoscopists may be comfortable with intermittent movement from the patient while others may only proceed if the patient is completely still. As mentioned, there were 82 endoscopists responsible for the 124 endoscopies included in our study; therefore, the variation in comfort with patient sedation may have contributed to the differences in the amount of propofol administered between groups.

The possibility exists that patients included in the cannabis non-users group were using cannabis but did not report use to their healthcare provider. Although the previously pejorative connotations associated with cannabis use are diminishing, some patients may fear the social or legal repercussions of disclosing cannabis use. This further emphasizes the need for providers to be educated on complementary and alternative therapies to accurately counsel patients and identify areas for management optimization. Due to the legal barriers to cannabis prescription, standardization of dose, route of administration, and strain were not possible, limiting the generalizability of our definition of cannabis use.

In summary, our study identified an association between cannabis use in patients with IBD with longer procedure time and a higher likelihood of exhibiting endoscopic inflammation. These correlations may be important in identifying the appropriate level of sedation and time allotment for endoscopic procedures in this population to optimize patient care and healthcare resource allocation. Future studies are needed to establish the role cannabis may have in IBD treatment as well as potential adverse effects and implications on patient outcomes.

## Data Availability

The data that support the findings of this study are available from the corresponding author, L.L., upon reasonable request.
